# A Gradient Dynamics-Based Singularity Avoidance Method for Backstepping Control of Underactuated TORA Systems

**DOI:** 10.3390/s24175458

**Published:** 2024-08-23

**Authors:** Changzhong Pan, Hongsen Pu, Zhijing Li, Jinsen Xiao

**Affiliations:** 1 School of Information and Electrical Engineering, Hunan University of Science and Technology, Xiangtan 411201, China; jgpshen@163.com; 2 School of Automation, Guangdong University of Petrochemical Technology, Maoming 525000, China; 3 Department of Mathematics, Guangdong University of Petrochemical Technology, Maoming 525000, China; 19209615227@163.com

**Keywords:** gradient dynamics, singularity, underactuated system, TORA, backstepping control, Lyapunov function

## Abstract

In this paper, a gradient dynamics-based control method is proposed to directly tackle the singularity problem in the backstepping control design of the TORA system. This method is founded upon the construction of an energy-like positive function, which includes an auxiliary variable in terms of the intermediate virtual control law. On this basis, a gradient dynamics is created to obtain a new virtual control command, which is capable of making the auxiliary variable gradually approach zero, thereby mitigating the issue of division by zero. The core innovation is the integration of the gradient dynamics into the recursive backstepping design to overcome the singularity problem and stabilize the system at the equilibrium quickly. In addition, it rigorously proves that all the signals in the closed-loop control system are uniformly ultimately bounded, and the tracking errors converge to a small neighborhood around zero through a Lyapunov-based stability analysis. Comparative simulations demonstrate that the proposed approach not only avoids the singularity issue, but also achieves a better transient performance over other methods.

## 1. Introduction

As a classical underactuated system [1,2,3,4], the translational oscillator with a rotational actuator (TORA) is a simplified model of dual-spin spacecrafts that was originally used to study the resonance capture phenomenon and the despin maneuver [5]. It is also used as a benchmark system for the design and performance test of various nonlinear control algorithms due to the characteristic of strong coupling and high nonlinearity [6,7]. From the point of application, the TORA system serves as an active mass damper (AMD) in engineering to suppressing vibrations in large-scale structures like super high-rise buildings, long-span bridges and offshore floating wind turbines [8,9,10]. Moreover, with appropriate modifications, it facilitates the investigation of self-synchronized phenomena in various mechanical systems such as vibration sifters, hands-held vibration tools, and vibration conveyors [11]. Therefore, the research of the TORA system holds considerable theoretical and practical significance. However, the TORA system is characterized by having fewer independent control inputs than the number of degrees of freedom, which makes the control design of the system extremely challenging.

To solve the challenging control problem, scholars have conducted extensive and in-depth research, resulting in the publication of numerous significant achievements in the past decades. The research varies between a focus on intelligent design methods and nonlinear design methods. Among the intelligent design methods, the methods based on fuzzy control [12,13,14,15,16,17] and neural network control [18,19] are proposed. However, these methods necessitate online learning processes, which result in the control algorithms having resource shortages and being challenging to implement in practical engineering applications. Among the nonlinear design methods, scholars have published a large number of control approaches according to the passive property of the TORA system and high nonlinearity, such as repetitive control [20], adaptive control [21,22], sliding mode control [23,24,25], passivity-based control [26,27,28,29,30,31], cascade-based control [32,33,34,35,36,37,38,39,40,41,42,43,44,45,46,47], etc.

It is noted that cascade-based control is the most extensively used method in the stabilization control of the TORA system. The key idea behind this method is to convert the model of the TORA system into a cascaded form by employing some coordinate transformations. At present, there are mainly two common transformation methods, one of which is the diffeomorphic transformation proposed by Bupp in [32] according to the geometric properties of the TORA system, while the other is the global coordinate transformation proposed by Olfati in [33], which is famous for underactuated systems. On the basis of these two transformations, feedback control laws can be derived by employing the backstepping technique, which is a powerful tool and recursive design methodology to study the globally asymptotic stabilization of nonlinear systems [48,49]. The key idea is to treat state variables as intermediate virtual control signals and to design control laws for them in sequence until the final actual control law is achieved. In [42], the TORA dynamics is transformed into a strict feedback cascade nonlinear system by employing the global coordinate transformation, and then an integral backstepping control method is proposed. In [43], a nonlinear dynamic surface controller is designed by inserting first-order filters into the backstepping design procedure to overcome the complexity explosion problem caused by repeated derivation of the virtual control signals in the backstepping procedure. In [44], a second-order command filter is introduced to solve the complexity explosion problem, and a filter error compensation dynamic system is designed to improve the control performance. In [45], a WNN-based adaptive backstepping control scheme is proposed to ensure the output of the TORA system to follow the desired trajectory in the presence of system uncertainties, and an experimental implementation of the nonlinear TORA system is introduced to verify the effectiveness and good performance of the control scheme. Some other types of backstepping controllers have been explored in [46,47]. Although the backstepping technique makes the cascade control design of the TORA system more convenient and efficient, there may exist singularities in the virtual control laws when the ball rotates across the horizontal position.

In order to solve the singularity problem, a switch control strategy is proposed in [47], where a nonlinear backstepping controller is designed for the system far away from the singularity, and a LQR linear controller is designed for the system around the singularity. The strategy of this singularity avoidance is simple and easy to implement, but the control signal is noncontinuous, which may lead to chattering problem and a long settling time. It is known that gradient descent is an iterative optimization method commonly used in machine learning and artificial intelligence to recursively approximate minimum deviation models. It is also appealing to use it to solve the singularity problem. In [50], Zhang neural dynamics and gradient-based neural dynamics are proposed to solve online nonlinear time-varying equations, and in [51], it is verified that the controllers based on Zhang dynamics (ZD) and gradient dynamics (GD) can conquer the singularity problem. Based on these findings, the GD is adopted for the stabilization of the TORA system in [52] to circumvent any singularities in the control law of maximal feedback linearization. Nevertheless, a global stability analysis of the closed-loop system integrated with the gradient dynamics is lacking.

Motivated by the above observations, this paper is aiming to directly tackle the singularity problem in the backstepping control design of the TORA system, and a nonsingular GD-based feedback controller is designed by incorporating the GD into the recursive backstepping procedure. Specifically, the dynamic model of the TORA system is transformed into a cascade system with strict feedback through the global coordinate transformation. Then, three virtual control laws and a real feedback control law are designed using the backstepping design technique. For the intermediate virtual control law with the singularity problem in the recursive procedure, an auxiliary variable is defined, and an energy-like function based on the GD method is designed. Finally, the stability of the developed control system is guaranteed through rigorous mathematical analysis, while numerical simulations are carried out along with comparisons to the existing approach to demonstrate the effectiveness and superiority of the proposed method. The innovation points of this paper are highlighted in the following three aspects.

(1) Different from the previous backstepping methods [43,44,45], this paper considers the singularity problem that may exist in the virtual control design when the ball rotates across the horizontal position, and a novel nonsingular control approach is presented by integrating the backstepping with a created gradient dynamics. To our knowledge, this is the first attempt to systematically design a GD-based backstepping controller for the stabilization of the TORA system.
(2) Unlike the singularity avoidance approaches in [47,52], the proposed controller is capable of generating a continuous control signal without any chattering phenomena. Furthermore, a rigorous proof and stability analysis of the whole closed-loop control system are given by using the Lyapunov theory.
(3) In comparison with the results in [39,45], the TORA system under the proposed controller can be stabilized at the equilibrium point with better control performance like a shorter settling time and a more reasonable control input.

The remainder of this paper is organized as follows. In Section 2, the description of the TORA system is presented, and the system model is transformed into a nonlinear feedback cascade form. The detailed design of the nonsingular backstepping controller with GD is described in Section 3. In Section 4, the global stability of the closed-loop control system is analyzed. Simulation results with comparisons are shown in Section 5, and conclusion remarks are finally given in Section 6.

## 2. Dynamic Model of the TORA System

**Figure 1:**
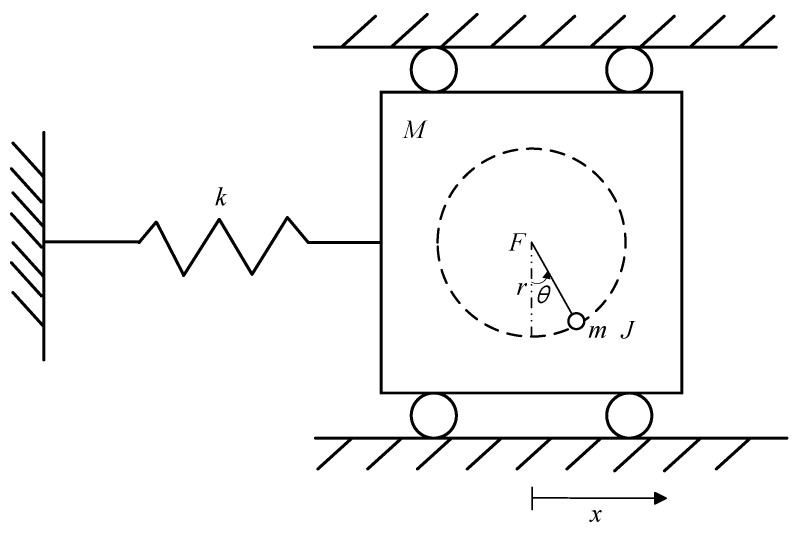
Physical model of the TORA system.

The physical model of the TORA system is shown in Figure 1, which consists of a cart and a ball. The ball with a mass of *m* and rotary inertia of *J* is installed on the cart and rotates with a radius of *r* under the action of the input torque of *F*, with an angle of rotation of θ. The cart with a mass of *M* is connected to the fixed end through a spring with an elastic coefficient of *k*, and performs one-dimensional linear motion on the horizontal plane, where the displacement is represented by *x*. It is seen that the input torque *F* controls both the rotation angle of the ball and the displacement of the cart, which makes it a typical underactuated system.

Using the Euler–Lagrange modeling method, the Lagrange equation of the TORA system can be expressed as

(1) $$\left\{\begin{array}{c} \frac{d}{dt}\left(\frac{\partial L}{\partial \dot{x}}\right)-\frac{\partial L}{\partial x}=0,\\ \frac{d}{dt}\left(\frac{\partial L}{\partial \dot{\theta }}\right)-\frac{\partial L}{\partial \theta }=F, \end{array}\right.$$

where *L* is the Lagrange function. The kinetic energy and potential energy of the TORA system are calculated as

(2) $$T=\frac{1}{2}M{\dot{x}}^{2}+\frac{1}{2}m{\dot{x}}^{2}+mr\dot{x}\dot{\theta }\cos\theta +\frac{1}{2}\left(mr^{2}+J\right){\dot{\theta }}^{2},$$

(3) $$V=\frac{1}{2}kx^{2}.$$

The Lagrange function is defined as

(4) $$L=T-V=\frac{1}{2}\left(M+m\right){\dot{x}}^{2}+mr\dot{x}\dot{\theta }\cos\theta +\frac{1}{2}\left(mr^{2}+J\right){\dot{\theta }}^{2}-\frac{1}{2}kx^{2}.$$

By substituting (4) into (1), the dynamic model of the TORA system can be expressed as [39,52]

(5) $$\left\{\begin{array}{c} \left(M+m\right)\ddot{x}+mr\ddot{\theta }\cos\theta -mr{\dot{\theta }}^{2}\sin\theta +kx=0,\\ mr\cos\theta \ddot{x}+\left(mr^{2}+J\right)\ddot{\theta }=F. \end{array}\right.$$

Notice from (5) that the TORA system possesses the property of underactuation since there is only one input torque *F* actuated on the ball, while the cart is indirectly controlled by the coupling relationship between the ball and the cart. This property makes the controller design for TORA system extremely difficult. In order to simplify the design of the controller, the coordinate transformation method described by Olfati-Saber in [33] is used to convert the system (5) into a cascade nonlinear system with strict feedback as [39]

(6) $$\left\{\begin{array}{c} z_{1}=x+\frac{mr}{M+m}\sin\theta ,\\ z_{2}=\left(M+m\right)\dot{x}+mr\dot{\theta }\cos\theta ,\\ z_{3}=\theta ,\\ z_{4}=\dot{\theta }. \end{array}\right.$$

Therefore, system (5) can be rewritten as

(7) $$\left\{\begin{array}{c} {\dot{z}}_{1}=\dot{x}+\frac{mr\dot{\theta }}{M+m}\cos\theta =\frac{z_{2}}{M+m},\\ {\dot{z}}_{2}=\left(M+m\right)\ddot{x}+mr\ddot{\theta }\cos\theta -mr{\dot{\theta }}^{2}\sin\theta =-kz_{1}+\varepsilon \sin z_{3},\\ {\dot{z}}_{3}=\dot{\theta }=z_{4},\\ {\dot{z}}_{4}=\ddot{\theta }=u, \end{array}\right.$$

where $\varepsilon =kmr/\left(M+m\right)$, and *u* is the control input of the system (7). From (5), it is not difficult to obtain the relationship between the control inputs *F* and *u*

(8) $$F=\alpha \left(\theta \right)u+\beta \left(\theta ,\dot{\theta }\right),$$

where

$$\alpha \left(\theta \right)=\left(mr^{2}+J\right)-\frac{{\left(mr\cos\theta \right)}^{2}}{M+m}>0,\beta \left(\theta ,\dot{\theta }\right)=-\frac{mr\cos\theta }{M+m}\left(kx-mr\sin\theta {\dot{\theta }}^{2}\right).$$

As can be seen from (7), the TORA system is transformed into a simpler cascade affine form, which makes the popular backstepping technique applicable for the control design. In addition, the coordinate transformation (6) is an invertible transformation, which is

(9) $$\left\{\begin{array}{c} x=z_{1}-\frac{mr}{M+m}\sin z_{3},\\ \theta =z_{3},\\ \dot{x}=\frac{z_{2}-mrz_{4}\cos z_{3}}{M+m},\\ \dot{\theta }=z_{4}. \end{array}\right.$$

Let $y={\left[x,\theta ,\dot{x},\dot{\theta }\right]}^{T}$ and $z={\left[z_{1},z_{2},z_{3},z_{4}\right]}^{T}$. By combining Equations (8) and (10), it can be concluded that y=0 is equivalent to z=0. Consequently, if a stabilizing controller *u* is designed for the system described in Equation (6), the controller *F* in Equation (7) guarantees the achievement of the stabilizing control objective stated in Equation (5).

## 3. Controller Design

This section is concerned with the nonsingular GD-based backstepping control (NGDBC) method for the system (7) such that all state variables of the TORA system are stabilized at the origin, even when it encounters singularity problems.

### 3.1. Stabilization Control Law

The backstepping design technique is used below to design a stabilization control law for system (7), and the design of the control law is divided into four steps.

**Step 1:** Define the error variable $e_{1}=z_{1}-z_{d}$, where $z_{d}$ is the desired trajectory. Since the objective is to stabilize the TORA system at the origin point, the desired trajectory $z_{d}$ is set as zero. Taking the time derivative of $e_{1}$ along (7) obtains

(10) $${\dot{e}}_{1}={\dot{z}}_{1}=\frac{z_{2}}{M+m}.$$

Choose the Lyaponov candidate function as $V_{1}=\frac{1}{2}{e_{1}}^{2}$, and then

(11) $${\dot{V}}_{1}=e_{1}{\dot{e}}_{1}=\frac{e_{1}z_{2}}{M+m}.$$

To stabilize $e_{1}$, a virtual control law $\alpha_{1}$ is defined for $z_{2}$. Define $e_{2}=z_{2}-\alpha_{1}$ as the discrepancy between the state $z_{2}$ and the virtual control $\alpha_{1}$. Based on (11), the virtual control law $\alpha_{1}$ is designed as

(12) $$\alpha_{1}=-k_{1}e_{1}\left(M+m\right),$$

where $k_{1}>0$. Substituting Equation (12) into Equation (11) obtains

(13) $${\dot{V}}_{1}=-k_{1}{e_{1}}^{2}-\frac{e_{1}e_{2}}{M+m}.$$

**Step 2:** Taking the time derivative of $e_{2}$ along (7) obtains

(14) $${\dot{e}}_{2}={\dot{z}}_{2}-{\dot{\alpha }}_{1}=-kz_{1}+\varepsilon \sin z_{3}-{\dot{\alpha }}_{1}.$$

Choose the Lyaponov candidate function as $V_{2}=V_{1}+\frac{1}{2}{e_{2}}^{2}$, and then

(15) $${\dot{V}}_{2}={\dot{V}}_{1}+e_{2}{\dot{e}}_{2}=-k_{1}{e_{1}}^{2}-\frac{e_{1}e_{2}}{M+m}+e_{2}\left(-kz_{1}+\varepsilon \sin z_{3}-{\dot{\alpha }}_{1}\right).$$

To stabilize $e_{2}$, a virtual control law $\alpha_{2}$ is defined for the term $\varepsilon \sin z_{3}$. Define $e_{3}=\varepsilon \sin z_{3}-\alpha_{2}$ as the discrepancy between the term $\varepsilon \sin z_{3}$ and the virtual control $\alpha_{2}$. Based on (15), the virtual control law $\alpha_{2}$ is designed as

(16) $$\alpha_{2}={\dot{\alpha }}_{1}+kz_{1}-k_{2}e_{2}+\frac{e_{1}}{M+m},$$

where $k_{2}>0$. Substituting Equation (16) into Equation (15) obtains

(17) $${\dot{V}}_{2}=-k_{1}{e_{1}}^{2}-k_{2}{e_{2}}^{2}+e_{2}e_{3}.$$

**Step 3:** Taking the time derivative of $e_{3}$ along (7) obtains

(18) $${\dot{e}}_{3}=\varepsilon {\dot{z}}_{3}\cos z_{3}-{\dot{\alpha }}_{2}=\varepsilon z_{4}\cos z_{3}-{\dot{\alpha }}_{2}.$$

Choose the Lyaponov candidate function as $V_{3}=V_{2}+\frac{1}{2}{e_{3}}^{2}$, and then

(19) $${\dot{V}}_{3}={\dot{V}}_{2}+e_{3}{\dot{e}}_{3}=-k_{1}{e_{1}}^{2}-k_{2}{e_{2}}^{2}+e_{2}e_{3}+e_{3}\left(\varepsilon z_{4}\cos z_{3}-{\dot{\alpha }}_{2}\right).$$

To stabilize $e_{3}$, a virtual control law $\alpha_{3}$ is defined for $z_{4}$. Define $e_{4}=z_{4}-\alpha_{3}$ as the discrepancy between the state $z_{4}$ and the virtual control $\alpha_{3}$. Based on (19), the virtual control law $\alpha_{3}$ is designed as

(20) $$\alpha_{3}=\frac{1}{\varepsilon \cos z_{3}}\left({\dot{\alpha }}_{2}-k_{3}e_{3}-e_{2}\right),$$

where $k_{3}>0$. Substituting Equation (20) into Equation (19) obtains

(21) $${\dot{V}}_{3}=-k_{1}{e_{1}}^{2}-k_{2}{e_{2}}^{2}-k_{3}{e_{3}}^{2}+\varepsilon e_{3}e_{4}\cos z_{3}.$$

**Step 4:** Taking the time derivative of $e_{4}$ along (7) obtains

(22) $${\dot{e}}_{4}={\dot{z}}_{4}-{\dot{\alpha }}_{3}=u-{\dot{\alpha }}_{3}.$$

Choose the Lyaponov candidate function as $V_{4}=V_{3}+\frac{1}{2}{e_{4}}^{2}$, and then

(23) $${\dot{V}}_{4}={\dot{V}}_{3}+e_{4}{\dot{e}}_{4}=-k_{1}{e_{1}}^{2}-k_{2}{e_{2}}^{2}-k_{3}{e_{3}}^{2}+e_{3}e_{4}\varepsilon \cos z_{3}+e_{4}\left(u-{\dot{\alpha }}_{3}\right).$$

Based on (23), the real control input *u* is finally designed as

(24) $$u={\dot{\alpha }}_{3}-k_{4}e_{4}-e_{3}\varepsilon \cos z_{3},$$

where $k_{4}>0$. Substituting Equation (24) into Equation (23) obtains

(25) $${\dot{V}}_{4}=-k_{1}{e_{1}}^{2}-k_{2}{e_{2}}^{2}-k_{3}{e_{3}}^{2}-k_{4}{e_{4}}^{2}\leq 0.$$

### 3.2. Singularity Avoidance Based on Gradient Dynamics

From Equation (20), it can be observed that when $z_{3}=\pm \frac{2n+1}{2}\pi \left(n=0,1,...\right)$, a division by zero occurs for $\alpha_{3}$, resulting in the singular value problem. This makes it challenging for the controller to achieve the desired control objective at the singular value point. To solve this issue, the gradient dynamics (GD) method is used to redesign the virtual control law $\alpha_{3}$. By multiplying both sides of Equation (20) simultaneously by $\cos z_{3}$ and then subtracting, an auxiliary variable is defined as

(26) $$\beta =\alpha_{3}\cos z_{3}-\frac{1}{\varepsilon }\left({\dot{\alpha }}_{2}-k_{3}e_{3}-e_{2}\right).$$

An energy-like function $\tau \left(\alpha_{3}\right)$ related to $\alpha_{3}$ is defined as

(27) $$\tau \left(\alpha_{3}\right)=\frac{1}{2}\beta^{2}.$$

Based on (27), the virtual control law $\alpha_{3}$, which is redesigned using the GD method, can be represented in the form of differential dynamics as

(28) $${\dot{\alpha }}_{3}=-\mu \frac{\partial \tau \left(\alpha_{3}\right)}{\partial \alpha_{3}}=-\mu \cos z_{3}\beta ,$$

where μ>0, which determines the rate of convergence of $\tau \left(\alpha_{3}\right)$ to the minimum point. To speed up the convergence, μ can be designed to be as large as possible within the range allowed by the control force output device.

**Remark** **1.** *According to Equation* (26)*, when β=0, Equation* (20)* is tenable. The concept behind the gradient dynamics approach is to design the dynamics in such a way that β gradually approaches zero, while also avoiding the issue of division by zero. It is important to note that $\tau \left(\alpha_{3}\right)$ is minimized if and only if β=0. Considering $\tau \left(\alpha_{3}\right)$ as a function of $\alpha_{3}$, adjusting $\alpha_{3}$ in the direction of the negative gradient of $\tau \left(\alpha_{3}\right)$ ensures that $\tau \left(\alpha_{3}\right)$ eventually reaches the minimum point.*

In order to facilitate the subsequent stability analysis, substituting Equation (26) into Equation (19) in **Step 3** obtains

(29) $$\begin{array}{cc} {\dot{V}}_{3} & =-k_{1}{e_{1}}^{2}-k_{2}{e_{2}}^{2}+e_{2}e_{3}+e_{3}\left[\varepsilon \left(e_{4}+\alpha_{3}\right)\cos z_{3}-{\dot{\alpha }}_{2}\right]\\ \; & =-k_{1}{e_{1}}^{2}-k_{2}{e_{2}}^{2}+e_{2}e_{3}+e_{3}\left(\varepsilon \beta -k_{3}e_{3}-e_{2}+\varepsilon e_{4}\cos z_{3}\right)\\ \; & =-k_{1}{e_{1}}^{2}-k_{2}{e_{2}}^{2}-k_{3}{e_{3}}^{2}+\varepsilon e_{3}e_{4}\cos z_{3}+\varepsilon e_{3}\beta . \end{array}$$

Subsequently, from (23), (24) and (29), it is easy to know that the time derivative of $V_{4}$ is

(30) $${\dot{V}}_{4}=-k_{1}{e_{1}}^{2}-k_{2}{e_{2}}^{2}-k_{3}{e_{3}}^{2}-k_{4}{e_{4}}^{2}+\varepsilon e_{3}\beta .$$

Combining the above design steps, the structure of the NGDBC designed in this paper is

(31) $$\left\{\begin{array}{c} \alpha_{1}=-k_{1}e_{1}\left(M+m\right),\\ \alpha_{2}={\dot{\alpha }}_{1}+kz_{1}-k_{2}e_{2}+\frac{e_{1}}{M+m},\\ \beta =\alpha_{3}\cos z_{3}-\frac{1}{\varepsilon }\left({\dot{\alpha }}_{2}-k_{3}e_{3}-e_{2}\right),\\ {\dot{\alpha }}_{3}=-\mu \cos z_{3}\beta ,\\ u={\dot{\alpha }}_{3}-k_{4}e_{4}-e_{3}\varepsilon \cos z_{3}. \end{array}\right.$$

**Figure 2:**
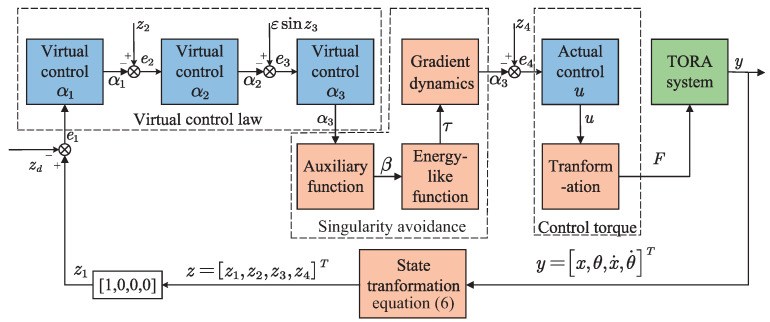
Block diagram of the proposed NGDBC scheme.

To summarize, the block diagram of the proposed NGDBC scheme is illustrated in Figure 2.

## 4. Stability Analysis

In this section, the convergence of the system error signals and the stability of the closed-loop control system under the proposed control method are analyzed using the Lyapunov stability theory. The main result is given by the following theorem.

**Theorem** **1.** *Consider the underactuated TORA system* (5)*. By selecting the appropriate values for $k_{i}\left(i=1,2,3,4\right)$ and μ in the backstepping controller* (31)*, it guarantees that all the signals in the closed-loop control system are uniformly ultimately bounded and the tracking errors converge to a small neighborhood around zero.*

**Proof.**  According to the analysis in Section 3.2 it can be seen that using the gradient dynamics method to redesign the virtual control law $\alpha_{3}$ is actually an asymptotic approximation to the optimal result of the virtual control law. Define the error between the virtual control law $\alpha_{3}$ and the optimal control law ${\alpha_{3}}^{*}$ as

(32) $$e_{\alpha_{3}}=\alpha_{3}-{\alpha_{3}}^{*}.$$

In order to analyze the influence of singularity on the stability of closed-loop system, the following two cases are analyzed, respectively.

**Case 1:**$\cos z_{3}\neq 0$, which means the singularity does not exist. Taking the time derivative of $e_{\alpha_{3}}$ along (28) obtains

(33) $$\begin{array}{cc} {\dot{e}}_{\alpha_{3}} & ={\dot{\alpha }}_{3}-{{\dot{\alpha }}_{3}}^{*}\\ & =-\mu \cos z_{3}\left[\alpha_{3}\cos z_{3}-\frac{1}{\varepsilon }\left({\dot{\alpha }}_{2}-k_{3}e_{3}--e_{2}\right)\right]-{{\dot{\alpha }}_{3}}^{*}\\ & =-\mu \cos^{2}z_{3}e_{\alpha_{3}}-{{\dot{\alpha }}_{3}}^{*}. \end{array}$$

Substituting Equation (32) into Equation (26) obtains

(34) $$\begin{array}{cc} \beta & =\left(\alpha_{3}+e_{\alpha_{3}}\right)\cos z_{3}-\frac{1}{\varepsilon }\left({\dot{\alpha }}_{2}-k_{3}e_{3}-e_{2}\right)\\ & ={\alpha_{3}}^{*}\cos z_{3}+e_{\alpha_{3}}\cos z_{3}-\frac{1}{\varepsilon }\left({\dot{\alpha }}_{2}-k_{3}e_{3}-e_{2}\right)\\ & =e_{\alpha_{3}}\cos z_{3}. \end{array}$$

Choose the Lyapunov function of the closed-loop system (7) and (31) as $V=V_{4}+\frac{1}{2}{e_{\alpha_{3}}}^{2}$. Taking the derivative of *V* and using Equations (33) and (34), it obtains

(35) $$\dot{V}=-k_{1}{e_{1}}^{2}-k_{2}{e_{2}}^{2}-k_{3}{e_{3}}^{2}-k_{4}{e_{4}}^{2}+\varepsilon e_{3}e_{\alpha_{3}}\cos z_{3}-\mu \cos^{2}z_{3}{e_{\alpha_{3}}}^{2}-{{\dot{\alpha }}_{3}}^{*}e_{\alpha_{3}}.$$

Obviously, there exists a positive real number ι such that $0<\iota ⩽\cos^{2}z_{3}⩽1$. Suppose that $|{\alpha_{3}}^{*}|⩽\kappa ,0<\kappa <+\infty$ exists during the control process. Then, Equation (35) can be simplified to

(36) $$\begin{array}{cc} \dot{V} & \leq -k_{1}{e_{1}}^{2}-k_{2}{e_{2}}^{2}-k_{3}{e_{3}}^{2}-k_{4}{e_{4}}^{2}+\varepsilon e_{3}e_{\alpha_{3}}+\left|e_{\alpha_{3}}\right|\left(-\mu \iota |e_{\alpha_{3}}|+\kappa \right)\\ \; & \leq -k_{1}{e_{1}}^{2}-k_{2}{e_{2}}^{2}-k_{3}{e_{3}}^{2}-k_{4}{e_{4}}^{2}+\varepsilon e_{3}e_{\alpha_{3}}-{\mu \iota e_{\alpha_{3}}}^{2}+\kappa \left|e_{\alpha_{3}}\right|. \end{array}$$

According to Young’s inequality, we know

(37) $$\left\{\begin{array}{c} \varepsilon e_{3}e_{\alpha_{3}}⩽\frac{\varepsilon }{2}\left({e_{3}}^{2}+{e_{\alpha_{3}}}^{2}\right),\\ \kappa |e_{\alpha_{3}}|⩽\frac{1}{2}\left(\kappa^{2}+{e_{\alpha_{3}}}^{2}\right). \end{array}\right.$$

Substituting Equation (37) into Equation (36) obtains

(38) $$\dot{V}\leq -k_{1}{e_{1}}^{2}-k_{2}{e_{2}}^{2}-\left(k_{3}-\frac{\varepsilon }{2}\right){e_{3}}^{2}-k_{4}{e_{4}}^{2}-\left(\mu \iota -\frac{\varepsilon +1}{2}\right){e_{\alpha_{3}}}^{2}+\frac{1}{2}\kappa^{2}.$$

Rewriting (38) into a compact form yields

(39) $$\dot{V}\leq -\chi V+\delta ,$$

where

(40) $$\left\{\begin{array}{c} \chi =\min\left(2k_{1},2k_{2},2k_{3}-\varepsilon ,2k_{4},2\mu \iota -\varepsilon -1\right),\\ \delta =\frac{1}{2}\kappa^{2}. \end{array}\right.$$

Select the design parameters $k_{3}>\varepsilon /2,\mu >\left(\varepsilon +1\right)/2\iota$ to ensure χ>0. Solving inequality (37) obtains

(41) $$V\left(t\right)\leq \frac{\delta }{\chi }+\left[V\left(0\right)-\frac{\delta }{\chi }\right]e^{-a_{0}t},$$

which means that $V\left(t\right)$ converges exponentially to the upper bound of δ/χ, i.e., as t→∞, $V\left(t\right)⩽\delta /\chi$. As a result, the system tracking error is ultimately bounded. Combining Equations (7) and (9), it can be seen that the system state variables are also bounded.

**Case 2:**$\cos z_{3}=0$, which means a singular point appears in the virtual control law of $\alpha_{3}$. From (28), it is known that ${\dot{\alpha }}_{3}=0$. Therefore, before and after the moment of singularity appears, we have $\alpha_{3}\left({t^{\prime }}_{-}\right)=\alpha_{3}\left(t^{\prime }\right)=\alpha_{3}\left({t^{\prime }}_{+}\right)$, and they are all bounded, where $t^{\prime }$ represents the moment when the singular point occurs; ${t^{\prime }}_{-}$ and ${t^{\prime }}_{+}$ indicate the previous moment and the later moment of $t^{\prime }$, respectively. In addition, bounded input $\alpha_{3}\left(t^{\prime }\right)$ at time $t^{\prime }$ causes the system state variables to be bounded as well. After the moment $t^{\prime }$, it returns to the situation discussed in Case 1.

Summarizing the above analysis, it is concluded that under the government of the proposed control law, regardless of whether a singular point occurs or not, all the signals in the closed-loop control system are uniformly ultimately bounded, and the tracking errors converge to a small neighborhood around zero. □

## 5. Simulation Results

In this section, two groups of numerical simulations are conducted to examine the performance of the NGDBC presented in this article. First, a comparative simulation with existing methods is performed to verify the superiority of the NGDBC. Then, parameter perturbations and external disturbance are imposed on the system to test the robustness of the proposed method.

All simulations are performed on MATLAB/Simulink 2022b, and the physical parameters of the TORA system given in [45] are utilized, i.e.,

(42) $$M=2.7\;\mathrm{kg},\;m=0.2\;\mathrm{kg},\;k=300\;N/m,\;r=0.18\;m,\;J=3\times 10^{-5}\;\mathrm{kg}\cdot m^{2}.$$

The parameters of the proposed controller in (31) are chosen as

(43) $$k_{1}=4,\;k_{2}=5,\;k_{3}=2,\;k_{4}=10,\;\mu =50.$$

### 5.1. Comparison Study

In order to better show the performance of the proposed method, a comparison study is carried out among the proposed NGDBC, the WNN-based adaptive backstepping controller (${\mathrm{ABC}}_{\mathrm{WNN}}$) [45], and the cascade-based controller (CBC) [39]. The detailed structures of the comparative controllers are omitted for brevity, and the control parameters are chosen as the same as those in [39,45] to ensure a fair comparison. Interested readers can refer to these references for details. For the purpose of comparison, the initial state ${\left[x\left(0\right),\;\theta \left(0\right),\;\dot{x}\left(0\right),\;\dot{\theta }\left(0\right)\right]}^{T}={\left[0.07,\;0,\;0,\;0\right]}^{T}$ is also chosen to be the same as [45].

**Figure 3:**
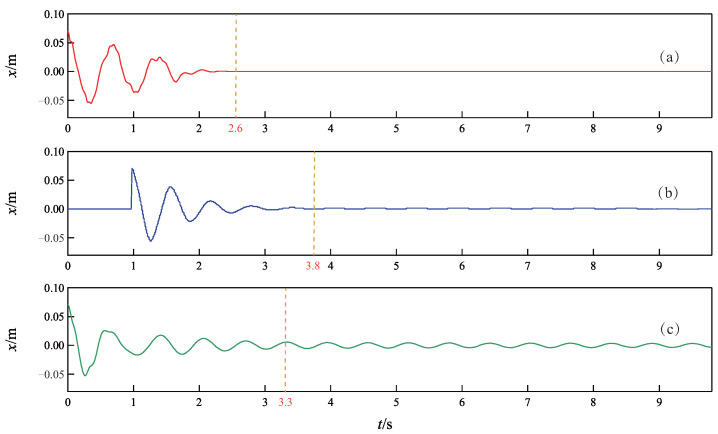
Time response curves of the cart position *x*. (**a**) Proposed method. (**b**) Method in [45]. (**c**) Method in [39].

**Figure 4:**
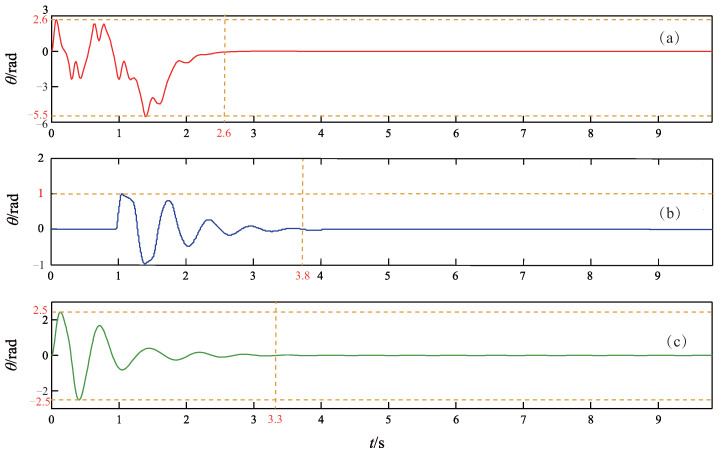
Time response curves of the ball angle θ. (**a**) Proposed method. (**b**) Method in [45]. (**c**) Method in [39].

**Figure 5:**
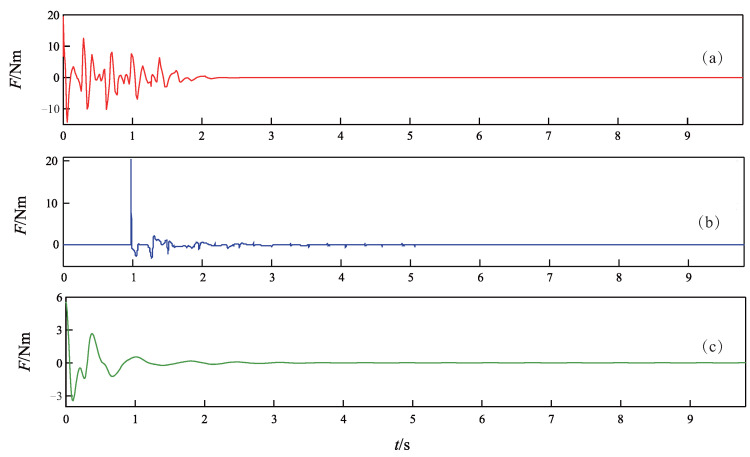
Time response curves of the control torque *F*. (**a**) Proposed method. (**b**) Method in [45]. (**c**) Method in [39].

The simulation results of the TORA under the three control methods are depicted in Figure 3, Figure 4 and Figure 5, where Figure 3 records the curves of the cart position, Figure 4 records the curves of the ball angle, and Figure 5 records the curves of the control torque. In addition, to quantitatively compare the performance of the three controllers, an index of settling time $t_{s}$ is defined as the shortest time when $x\left(t\right)$ and $\theta \left(t\right)$ enter the ranges of $|x\left(t\right)|⩽0.03$ m and $|\theta \left(t\right)|⩽0.087$ rad, respectively, and they never deviate from these ranges thereafter [7].

As can be seen from Figure 3, Figure 4 and Figure 5, all three controllers are effective to drive the cart and ball to rest at the equilibrium point. However, the proposed controller achieves faster control performance than the other two controllers. More precisely, the settling time $t_{s}$ is 2.6 s for the proposed NGDBC, 3.8 s for the ${\mathrm{ABC}}_{\mathrm{WNN}}$, and 3.3 s for the CBC. Although the maximum amplitude of the ball angle in Figure 4b is the smallest, the output of the controller in Figure 5b is characterized by a large amplitude control signal, often saturating during the initial 1 s, with substantial high-frequency content [45], which results in a long settling time. The simulation results in this group demonstrate that the transient performance of the proposed control scheme is superior to the ${\mathrm{ABC}}_{\mathrm{WNN}}$ and the CBC methods.

### 5.2. Robustness Test

In order to test the robustness of the proposed method, the external disturbance and several parameter perturbations are imposed on the system. The external disturbance is a time-varying sinusoid disturbance, d(t)=sin(4πt), which is added from 0 s to 10 s. The parameter perturbations are ▵M=+10%M,▵m=+10%m,▵J=−10%J,▵k=−10%k. The initial state is chosen as ${\left[x\left(0\right),\;\theta \left(0\right),\;\dot{x}\left(0\right),\;\dot{\theta }\left(0\right)\right]}^{T}={\left[0.2,\;0,\;0,\;0\right]}^{T}$.

**Figure 6:**
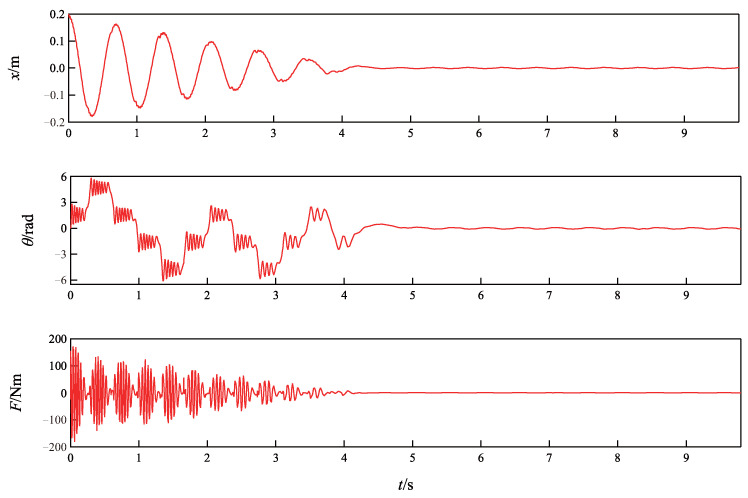
Simulation results of the TORA system with external disturbances and parameter uncertainties.

The results of the simulation are presented in Figure 6. It is observed from Figure 6 that the cart and the ball can still be driven to the neighborhood of the origin even there exist parameter perturbations and vibration. The simulation results in this group verify that the proposed NGDBC has good robustness to external disturbances and parameter uncertainties. However, after repeated tests, it is found that the robustness of the proposed controller is limited, i.e., the cart and the ball will not be able to converge to a neighborhood of the origin when vibration or parameter perturbations over 20% are imposed.

## 6. Conclusions

This paper proposed a novel gradient dynamics-based control method to address the singularity issue in the recursive backstepping design of TORA system. An energy-like positive function, which introduced an auxiliary variable in terms of intermediate virtual control law, was built to facilitate the incorporation of gradient dynamics into the backstepping design procedure. A rigorous stability of the closed-loop control system was analyzed using Lyapunov stability theory, and it proved that all the signals were uniformly ultimately bounded, and the errors converged to a small neighborhood around zero. Simulation results with comparisons were presented to show that the method was effective in avoiding any singularities in the whole control process and superior to other methods in aspects of transient and steady-state performance.

It is worth mentioning that although the singularity problem is tackled in the proposed approach, the external disturbances and the internal parameter uncertainties still have great influence on the control performance. Therefore, the stabilization of the TORA system with uncertainties is still challenging and needs to be further investigated in future work.

## Data Availability

Data are contained within the article.
